# Local and National Advocacy Support

**DOI:** 10.1007/s10875-012-9736-6

**Published:** 2012-07-19

**Authors:** Ralph S. Shapiro, Marcia Boyle, Elena E. Perez

**Affiliations:** 1 Midwest Immunology Clinic, Plymouth, MN USA; 2 Immune Deficiency Foundation, 40 West Chesapeake Avenue, Suite 308, Towson, MD 21204 USA; 3 University of South Florida, All Children’s Hospital, St Petersburg, FL USA

**Keywords:** Primary immunodeficiency disease, treatment guidelines, Immune Deficiency Foundation, AAAAI IGIV toolkit, Jeffrey Modell Foundation

## Abstract

Decisions by third-party payors that are restricting delivery of appropriate IgG treatment for primary immunodeficiency disease (PIDD) are summoning action from patients, physicians, and their organizations to ensure that high quality treatment remains accessible. Some of the strongest advocacy to date is from patient organizations, such as the Immune Deficiency Foundation (IDF), which strive to educate stakeholders on key issues that determine patient access to appropriate IgG treatment. These issues include the ability to choose the appropriate site of care based on a patient’s experience and circumstance and greater awareness of product choice. Advocacy by physicians on these issues at the local level is needed, as are national efforts by organizations such as the American Academy of Allergy, Asthma & Immunology and their regional societies.

## Introduction

The preceding article in this supplement provided clear support for the view that policy decisions by third-party payors restrict delivery of appropriate immunoglobulin G (IgG) treatment for patients with primary immunodeficiency disease (PI) and that effective action to reverse these policies requires coordinated actions by patients, physicians, and advocacy groups. Initiatives at both the local and national levels are important for ensuring that patients with PI retain or, in some cases, regain access to appropriate care with IgG. This article reviews advocacy resources and activities undertaken at the national and local levels that have improved appropriate access to care.

## Advocacy Resources

### Treatment Guidelines

Treatment guidelines for the management of patients with PI provide an important basis for advocacy efforts. These recommendations establish the standard of care for PI patients and are thus important tools for challenging insurance company policies that are inconsistent with evidence-based medicine. The most prominent guideline in the United States is "The use of intravenous immunoglobulin and adjunctive therapies in the treatment of primary immunodeficiencies: A working group report of and study by the Primary Immunodeficiency Committee of the American Academy of Allergy, Asthma & Immunology (AAAAI)" [1]. These guidelines are currently under revision, and an update will be submitted in 2012. The Canadian Blood Services and National Advisory Committee has published a similar comprehensive document highlighting the use of IgG therapies for PI [2].

### The AAAAI Intravenous Ig (IGIV) Toolkit

The AAAAI IGIV Toolkit, an adjunct to the treatment guidelines published by this group, is another important resource that can be used for educating insurers and regulators; it also provides information for physicians to help ensure safe and effective IGIV administration. The development of this Toolkit was stimulated by anonymous results from an AAAAI survey indicating that >95 % of respondents believed that current reimbursement standards posed a risk to the health of their PI patients [3]. This Toolkit was approved by the AAAAI Board of Directors, the Clinical Immunology Society, and the Immune Deficiency Foundation (IDF); it provides an example of how these national organizations can work together to achieve an important common goal.

The AAAAI IGIV Toolkit sets forth 8 guiding principles for the safe, effective, and appropriate use of IGIV for PI (Table I) [4]. Although the Toolkit was not developed exclusively to educate insurers, it does address key areas in which they have created barriers to care for PI patients. The Toolkit emphasizes that IGIV treatment is appropriate for patients with PI, improves quality of life, and may even be lifesaving. It also states that IGIV treatment should not be interrupted once a definitive PI diagnosis has been established. The Toolkit provides guidance regarding IGIV dosing. The generally accepted IGIV starting dose is 400–600 mg/kg every 3–4 weeks, and the route of IgG administration must be based upon patient characteristics and patient preference [4]. It should be noted that the use of IGSC has been increasing in the US in recent years.

**Table I:** Principles in the AAAAI IGIV Toolkit [4]

1. **Indication** - IGIV therapy is indicated as replacement therapy for patients with PI characterized by absent or deficient antibody production. This is an FDA-approved indication for IGIV for which all currently available products are licensed.
2. **Diagnoses** - There are a large number of PI diagnoses for which IGIV is indicated and recommended. Many have low total levels of IgG, but some have a normal level with documented specific antibody deficiency.
3. **Frequency of IGIV treatment** - IGIV is indicated as continuous replacement therapy for primary immunodeficiency. Treatment should not be interrupted once a definitive diagnosis has been established.
4. **Dose** - For patients with primary immunodeficiency, IGIV is indicated at a starting dose of 400–600 mg/kg every 3–4 weeks. Less frequent treatment, or use of lower doses, is not substantiated by clinical data.
5. **IgG trough levels** - IgG trough levels can be useful in some diagnoses to guide care but are NOT useful in many and should NOT be a consideration in access to IGIV therapy.
6. **Site of care** - The decision to infuse IGIV in a hospital, hospital outpatient, community office, or home-based setting must be based upon the clinical characteristics of the patient.
7. **Route** - Route of immunoglobulin administration must be based upon patient characteristics. The majority of patients are appropriate for intravenous, while a subset is appropriate for subcutaneous therapy.
8. **Product** - IGIV is not a generic drug, and IGIV products are not interchangeable. A specific IGIV product needs to be matched to patient characteristics to ensure patient safety. A change of IGIV product should occur only with the active participation of the prescribing physician.

Adapted from the Primary Immunodeficiency Committee of the American Academy of Allergy, Asthma and Immunology [4].

The Toolkit also challenges the concept that a single IgG product can be used for all patients and that formulary restriction to control costs is a rational approach to care. The Toolkit's guiding principles state that IGIV is not a generic drug and that IGIV products are not interchangeable. A specific IGIV product needs to be matched to patient characteristics to ensure safety. Also, a change of IGIV product should occur only with the active participation of the prescribing physician [4].

In addition to the 8 principles, the Toolkit contains background information, a detailed explanation of each principle, a position paper on the use of IGIV, the current AAAAI guidelines, and site-of-care treatment recommendations. Also included is a template for letters to contact medical directors advocating coverage for IGIV. The IGIV Toolkit is currently being revised with the aim of including more information about IGSC and the risks associated with product switching.

## Case Studies: National and Local Intervention

### National Advocacy: Restrictive Formulary for IgG Products

In December 2010, a major insurance company issued a special bulletin stating that IgG products were considered clinically equivalent and interchangeable and that, effective April 1, 2011, a formulary for IgG with only one preferred product for both IGIV and IGSC would be instituted. To access a nonpreferred IgG product, a patient must have a documented drug therapy failure with the preferred product. The IDF Medical Advisory Committee (MAC) responded to this action with a product choice resolution stating that 1) IgG therapies are not generic; 2) a policy that provides access to alternate therapies only after a patient has suffered an adverse event is unacceptable; 3) the incidence of adverse reactions increases when a patient changes therapies; and 4) not all brands of IgG will always be immediately available, and restricting physicians to a single approved product could lead to delays in treatment [5]. IDF sent a letter and the IDF MAC resolution to the insurer and initiated discussions with them.

Because April 1 was less than a month away and IDF had heard of no official changes to the initial policy, IDF launched a campaign on March 16, 2011 with a press release in local markets and the launch of a blog. IDF reached out to other patient organizations and local medical groups for support and was joined by the Pennsylvania Allergy and Asthma Association and the Pennsylvania Medical Society. In addition, IDF identified patients in the community who would be impacted by the policy and who could mobilize with the media campaign.

On March 18, 2011, the insurance company responded to these actions by making the following changes in its "fail-first" policy for switching from the preferred IgG: 1) patients who had an adverse reaction to the preferred product in the past were not required to try it again; 2) patients who had medical evidence that a switch to the preferred product would likely cause an adverse reaction due to product characteristics (e.g., sugar content, IgA content, sodium content) are not required to use it; 3) patients with documented objective laboratory evidence (e.g., IgA level) that would predispose them to significant adverse events were not required to use the preferred product; and 4) patients <18 years of age who were currently stable on a nonpreferred product were not required to switch.

IDF responded to these changes by stating that, although the changes were a good step forward, they did not provide sufficient protection for adult patients already stabilized on an IgG therapy that was not the insurer’s preferred brand. IDF initiated a call to action that resulted in 650 letters being sent to the insurance company’s manager. On March 28, 2011, the insurer stated that it would “grandfather,” or continue to cover, all IgG products not on its preferred formulary for current health plan members with PI. For new members who join the health plan and receive an IgG product other than the preferred product, the insurer will individually evaluate each case based on clinical rationale.

### Local Advocacy: Clinical Care Guidelines that Impacted Access to Care for Patients

In March 2009, a large health plan in Minnesota began denying claims for IgG for a number of PI patients. This prompted an IDF-led letter-writing campaign to the health plan, with copies to the governor, the state attorney general, and the insurance commissioner. In March 2010, the Midwest Immunology Center in Minnesota withdrew from the health plan network in protest of their policies; this resulted in patients being sent to in-network physicians, some of whom had little, if any, experience in the management of patients with PI. Members of the IDF MAC and AAAAI PI Committee jointly reviewed the health plan guidelines and sent a letter pointing out ways in which the policy did not reflect the standard of care recommended by the AAAAI and was limiting access to care. The cooperative effort between the IDF MAC, the AAAAI, and the health plan led to the AAAAI deciding to update their IgG guidelines, with the health plan ultimately updating their policies in April 2011.

## Lessons Learned

Experience from these and other cases has provided important lessons for avoiding and responding to problems of appropriate access to IgG. Problems with treatment approval may be avoided by providing the appropriate diagnosis (e.g., common variable immunodeficiency, rather than hypogammaglobulinemia or dysgammaglobulinemia) so that it may be properly coded for the insurance provider, as well as by including pertinent clinical and laboratory information in a submission packet. Inclusion of relevant medical literature will speed prior authorization. If authorization is denied, it is important to gain access to an insurance company medical director who can competently discuss the relevant medical issues. If this fails, a peer-to-peer review can be requested in which a physician who treats patients with immunodeficiency may be included in the process. It is very important to have information regarding current standards of care available to support a request for treatment (e.g., AAAAI Committee on Immune Deficiency guidelines on immunoglobulins [1]).

The results from the case studies summarized in the preceding section underscore the importance of both local and national advocacy groups to force changes in insurer policies that negatively affect patient care. Local advocacy may include actions (e.g., letter-writing campaigns, telephone campaigns) by family groups affiliated with the IDF and by the Jeffrey Modell Foundation (JMF), a global nonprofit organization dedicated to early diagnosis, meaningful treatments, and, ultimately, cures for PI achieved through research, physician education, public awareness, advocacy, patient support, and newborn screening [6]. In addition, supporting local legislation may be helpful in forcing insurance companies to evaluate their policies and change those that do not conform to best medical practices. For example, legislation proposed in Minnesota (Minnesota Statutes, chapters 62Q; 151; Coverage Of Plasma Protein Therapies And Home Nursing Services) states that any health plan offered by a health plan company must carry all FDA-approved plasma protein therapies in the formulary, provide coverage for home nursing services, complete pre-authorization within 24 h or one business day, and provide the option of receiving covered services at more than one pharmacy. In addition, this legislation states that the Board of Pharmacy shall promulgate rules that include standards established by the MACs of the patient groups and professional societies representing individuals with PI.

National advocacy may include actions by the IDF and its MAC, the AAAAI Committee on PI, and public awareness campaigns sponsored by the IDF and/or the JMF.

## Conclusions

Physicians and other healthcare professionals involved in the care of patients with PI should be aware of policies that limit access to appropriate care. It is often difficult for individual providers to effectively combat insurer decisions that limit treatment, but local, regional, and national resources are available to help address these problems. It is important for healthcare professionals and advocacy groups to monitor regulation and legislation aimed at governing insurance companies and to intervene when needed to make policies consistent with current standards of care.

## Discussion

Questions for the audience at the symposium underscored the difficult decisions insurers impose on physicians. For example, physicians may be required to repeatedly demonstrate that patients indeed have PI in order to gain reauthorization for treatment. Symposium attendees were given the following scenario: "The patient tells you that their IGIV product is being switched to another ‘brand’ that has been previously a problem for him or her. What would you do?" Nearly 90 % of 54 respondents stated that they would pull the documentation of the tolerability issue on the previous product to discuss with the insurance medical director. This suggests that many attendees at the symposium understood the best approach to negotiation in these situations.
